# Travel-acquired infections and illnesses in Canadians: surveillance report from CanTravNet surveillance data, 2009–2011

**Published:** 2014-02-11

**Authors:** Andrea K Boggild, Jennifer Geduld, Michael Libman, Brian J Ward, Anne E McCarthy, Patrick W Doyle, Wayne Ghesquiere, Jean Vincelette, Susan Kuhn, David O Freedman, Kevin C Kain

**Affiliations:** Andrea K. Boggild, MSc, MD, DTMH, FRCPC, is the Clinical Director of the Tropical Disease Unit and a Staff Physician in the Division of Infectious Diseases, University Health Network – Toronto General Hospital; Assistant Professor, Department of Medicine, University of Toronto; and the Parasitology Lead with Laboratory Services, Public Health Ontario, Toronto, Ontario.; Jennifer Geduld, MHSc, BSc, is Manager, Epidemiology with the Travel and Migration Health Division, Infectious Disease Prevention and Control Branch, Public Health Agency of Canada, Ottawa, Ontario.; Michael Libman, MD, is Director of the Centre for Tropical Diseases, Director of the Division of Infectious Diseases, and member of the Department of Microbiology, McGill University Health Centre, Montreal, Quebec.; Brian J. Ward, MSc, MDCM, DTM&H, is Professor of Medicine and Microbiology, Division of Infectious Diseases, Department of Microbiology, McGill University Health Centre, Montreal, Quebec.; Anne E. McCarthy, MD, FRCPC, DTM&H, is Professor of Medicine, University of Ottawa, and Director of the Tropical Medicine and International Health Clinic, The Ottawa Hospital–General Campus, Ottawa, Ontario.; Patrick W. Doyle, MD, MHSc, FRCPC, is Medical Microbiologist with the Division of Medical Microbiology and Infection Control, Vancouver General Hospital, and Clinical Professor, Department of Pathology and Laboratory Medicine, University of British Columbia, Vancouver, British Columbia.; Wayne Ghesquiere, MD, FRCPC, is an Infectious Diseases and Internal Medicine Consultant and Section Chief of Infectious Diseases with the Vancouver Island Health Authority, Victoria, British Columbia, and Clinical Assistant Professor of Medicine, University of British Columbia, Vancouver, British Columbia.; Jean Vincelette, MD, is Full Clinical Professor with the Département de microbiologie médicale et infectiologie, Hôpital Saint-Luc, Centre hospitalier de l'Université de Montréal, Montreal, Quebec.; Susan Kuhn, MD, is Head, Section of Pediatric Infectious Diseases, and Associate Professor, Departments of Pediatrics and Medicine, University of Calgary, Calgary, Alberta.; David O. Freedman, MD, is Professor of Medicine and Epidemiology and Director of the UAB Travelers Health Clinic with the Gorgas Center for Geographic Medicine, Division of Infectious Diseases, University of Alabama at Birmingham, Birmingham, Alabama.; Kevin C. Kain, MD, FRCPC, is Co-Director of the Tropical Disease Unit and a Professor of Medicine, Division of Infectious Diseases, Department of Medicine, University Health Network – Toronto General Hospital and University of Toronto, and Director of the Sandra A. Rotman Laboratories, Sandra Rotman Centre, University Health Network, Toronto, Ontario.

## Abstract

**Background:**

Important knowledge gaps exist in our understanding of migration medicine practice and the impact of pathogens imported by Canadian travellers. We present here a comprehensive, Canada-specific surveillance summary of illness in a cohort of returned Canadian travellers and new immigrants.

**Methods:**

We extracted and analyzed (using standard parametric and nonparametric techniques) data from the Canadian Travel Medicine Network (CanTravNet) database for ill returned Canadian travellers and new immigrants who presented to a Canadian GeoSentinel Surveillance Network site between September 2009 and September 2011.

**Results:**

During the study period, 4365 travellers and immigrants presented to a CanTravNet site, 3943 (90.3%) of whom were assigned a travel-related diagnosis. Among the 3115 non-immigrant travellers with a definitive travel-related diagnosis, arthropod bite (n = 127 [4.1%]), giardiasis (n = 91 [2.9%]), malaria (n = 77 [2.5%]), latent tuberculosis (n = 73 [2.3%]), and strongyloidiasis (n = 66 [2.1%]) were the most common specific etiologic diagnoses. Among the 828 immigrants with definitive travel-related diagnoses, the most frequent etiologies were latent tuberculosis (n = 229 [27.7%]), chronic hepatitis B (n = 182 [22.0%]), active tuberculosis (n = 97 [11.7%]), chronic hepatitis C (n = 89 [10.7%]), and strongyloidiasis (n = 41 [5.0%]). Potentially serious infections, such as dengue fever (61 cases) and enteric fever due to *Salmonella enterica* serotype Typhi or Paratyphi (36 cases), were common. Individuals travelling for the purpose of visiting friends and relatives (n = 500 [11.6% of those with known reason for travel]) were over-represented among those diagnosed with malaria and enteric fever, compared with other illnesses (for malaria 34/94 [36.2%] v. 466/4221 [11.0%]; for enteric fever, 17/36 [47.2%] v. 483/4279 [11.3%]) (both *p* < 0.001). For cases of malaria, there was also overrepresentation (compared with other illnesses) from business travellers (22/94 [23.4%] v. 337/4221 [8.0%]) and males (62/94 [66.0%] v. 1964/4269 [46.0%]) (both *p* < 0.001). Malaria was more likely than other illnesses to be acquired in sub-Saharan Africa (*p* < 0.001), whereas dengue was more likely than other illnesses to be imported from the Caribbean and South East Asia (both *p* = 0.003) and enteric fever from South Central Asia (24/36 [66.7%]) (*p* < 0.001).

**Interpretation:**

This analysis of surveillance data on ill returned Canadian travellers has detailed the spectrum of imported illness within this cohort. It provides an epidemiologic framework for Canadian practitioners encountering ill returned travellers. We have confirmed that travel to visit friends and relatives confers particularly high risks, which underscores the need to improve pretravel intervention for a population that is unlikely to seek specific pretravel advice. Potentially serious and fatal illnesses such as malaria and enteric fever were common, as were illnesses of public health importance, such as tuberculosis and hepatitis B.

Canadians represent an increasingly mobile population. The more affordable nature of air travel, the globalization of trade and commerce, the greater representation of developing-world immigrants within the Canadian population, and a trend toward "voluntourism" and ecotourism have all contributed to a greater number of Canadians crossing international borders than ever before. The stereotypical beachdestination vacationer is increasingly being replaced with off-the-beaten-path backpackers, new Canadian immigrants and their family members returning home to visit friends and relatives, last-minute business travellers, and researchers, missionaries, and volunteers heading to ever more exotic locales. This paradigm shift is supported by data from the World Tourism Organization and Statistics Canada. For example, in 2011, Canadians spent US$33 billion on international tourism, up from US$29.6 billion in 2010.^1^ Along with traditional destinations such as the United States, the United Kingdom, and France, tropical and developingworld destinations, including Mexico, Cuba, and the Dominican Republic, are among the top 10 foreign destinations chosen by Canadian travellers.^2^

Travelling to the developing world necessarily puts travellers and migrants at risk for communicable infectious diseases, with 20%–70% of returned travellers suffering some sort of illness.^3^–^5^ Although single-centre studies in other countries and multinational studies of travel-acquired illness have been conducted, a comprehensive multicentre comparison of the spectrum of illnesses acquired by a broad range of Canadian travellers returning from regions on all continents has been lacking. Understanding of the range and frequency of infectious diseases in Canadian travellers is based primarily on existing synthesized knowledge of travelacquired illness in other populations. Expert references such as the World Health Organization's *International Travel and Health*^6^ and the so-called "Yellow Book" published by the US Centers for Disease Control and Prevention,^7^ as well as the Public Health Agency of Canada's Committee to Advise on Tropical Medicine and Travel (CATMAT; www.phac-aspc.gc.ca/tmp-pmv/catmat-ccmtmv/index-eng.php), provide guidance to practitioners, yet data specifically focusing on Canadian travellers in support of recommendations from these sources are lacking. Furthermore, although many imported communicable diseases are designated as nationally notifiable to the Public Health Agency of Canada,^8^ the quality of data accrued is hindered by delayed reporting and underreporting, which have led to "an incomplete picture of the incidence of communicable diseases in Canada."^9^

We synthesized Canada-specific surveillance data on ill returned travellers with the goal of informing provincial- and national-level public health policy and strategic initiatives to reduce the incidence of preventable infections, increase the efficiency of public health infrastructure, and improve the uptake of preventive pretravel care. In addition, this analysis provides accurate epidemiologic data about travel-associated infectious diseases in travellers returning to Canada, which are necessary to guide clinical decision-making by front-line Canadian practitioners.

## Methods

### Data source

Surveillance data were collected using the GeoSentinel Global Surveillance Network platform. This network comprises 57 specialized travel and tropical medicine clinics on 6 continents, which contribute anonymous, delinked clinician- and questionnairebased travel surveillance data on all ill travellers examined to a centralized Structured Query Language database^10^,^11^ (for additional details, see www.geosentinel.org). The GeoSentinel data-collection protocol is reviewed cyclically by the institutional review board officer at the National Center for Emerging and Zoonotic Infectious Diseases at the US Centers for Disease Control and Prevention and is classified as public health surveillance, not human-subjects research requiring submission to and approval from institutional review boards. Canada currently has 6 sites in 4 provinces belonging to the global GeoSentinel Global Surveillance Network, which have grouped together as the core sites of CanTravNet, the Canadian Travel Medicine Network.

The 6 Canadian sites are large referral-based outpatient clinics that primarily serve the Greater Vancouver, Victoria, Calgary, Toronto, Ottawa, and Montreal areas, which together account for 47% of the Canadian population (or a catchment of about 15.5 million people). They are staffed by specialists in travel and tropical medicine and are typically secondary or tertiary points of care for patients, although immediate referral from the emergency departments attached to the respective parent hospitals is common. All of the centres provide post-travel services, which are billed under the provincial health plans. Collected data include each patient's demographic characteristics, detailed recent travel itinerary, all countries visited within the past 5 years, reason for travel, affected organ system, and whether the patient had a pretravel encounter with a health care provider. Final diagnoses are made by the attending physicians at each CanTravNet site, and each diagnosis is assigned a diagnostic code selected from a standardized list of more than 500 diagnostic entities, some of which are etiologic (e.g., *Campylobacter*) and others of which are syndromic (e.g., acute diarrhea). Syndromic codes are entered when an etiologic code cannot be assigned because of use of empiric therapy, self-limited disease, or inability to justify complete or sophisticated work-up as part of routine clinical practice. All CanTravNet sites contribute microbiologically confirmed data, where available, based on the best reference diagnostic tests (including serologic assays and polymerase chain reaction) available in Canada at the time. "Probable" diagnoses are restricted to patients with pathognomonic physical findings (e.g., tick eschar), clinical response to highly specific therapy, or classical presentation and exposure history with laboratory exclusion of other possible etiologies.

### Definitions

Six possible reasons for most recent travel are available in GeoSentinel: immigration, including immigration by refugees; tourism; business; missionary, volunteer, research, or aid work; visiting friends and relatives; and "other," which includes travel for education, military service, and "medical tourism." Those whose reason for travel is listed as "immigration" include patients whose post-travel diagnosis is related to their emigration travel or long-term residence in the home country, rather than a particular isolated international trip.^11^ Examples of diagnoses attributable to immigration-related travel are leprosy, tuberculosis, and certain chronic helminthic infections, such as hydatid disease or neurocysticercosis, as well as diseases imported by new immigrants to Canada. Travel to visit friends and relatives is defined as travel by an immigrant who is ethnically and/or racially distinct from the majority population in his or her current country of residence and who is returning to his or her homeland to visit friends and relatives. This type of travel also refers to travel by children of foreign-born parents (i.e., second-generation immigrants) who return to their parents' homeland to visit friends and relatives. The term "visiting friends and relatives" is typically applied to individuals travelling from a high-income country of current residence to a low-income country of origin.^12^ "Medical tourists" are defined as those whose primary purpose of travel is to seek emergency or elective care and who, as a consequence of the travel, acquire an infectious complication secondary to the medical care received or become ill with an infectious or noninfectious disease while abroad.

Countries of exposure and travel were assigned to 1 of 14 regional classifications:^11^ North America, Central America, the Caribbean, South America, Western Europe, Eastern Europe, the Middle East, North Africa, sub-Saharan Africa, South Central Asia, South East Asia, North East Asia, Australia/New Zealand, and Oceania.

### Inclusion criteria

We extracted and analyzed demographic, clinical, and travel-related data on Canadian citizens and new immigrants to Canada encountered after completion of their international travel or residence abroad and seen from September 2009 to September 2011 at any of 5 CanTravNet sites that were in operation at that time. We included only patients with probable or confirmed final diagnoses (specific etiologic or syndromic diagnoses, as described above). The term "cohort of travellers" refers to the entire cohort of travellers encountered at CanTravNet sites, including immigrants. "Ill returned travellers" refers to travellers or immigrants within the larger cohort who were deemed to have a definitive "travel-related" diagnosis, as opposed to diagnoses unrelated to travel or not ascertainable.

### Statistical analysis

Extracted data were managed in a Microsoft Access database and were analyzed with standard parametric and nonparametric techniques. Data for categorical variables were compared with Yates' corrected χ^2^ analysis, and data for continuous variables were analyzed for significant differences with the Student *t* test or, in the case of non-normally distributed parameters, the Mann–Whitney rank sum test. Differences between groups of continuous variables were compared using one-way analysis of variance (ANOVA) or Kruskal–Wallis one-way ANOVA on ranks. All statistical tests were 2-sided. Statistical computations were performed with SigmaStat 2.03 software (SPSS Inc., Chicago, IL). The level of significance was set at *p* < 0.05.

## Results

### Patients and demographic characteristics

For the surveillance period covered by this analysis, 4365 travellers presented to a CanTravNet site and were assigned totals of 4776 confirmed and 535 probable diagnoses. Among these 4365 travellers seen, 3943 (90.3%) had a definitive travel-related diagnosis (hereafter referred to as "ill returned travellers," as defined above), 363 (8.3%) had a non-travel-related diagnosis, and 59 (1.4%) had a diagnosis for which relation to travel could not be ascertained. Each of the 4365 travellers presented to 1 of 5 CanTravNet sites as follows: 1899 (43.5%) to Montreal— McGill, 1088 (24.9%) to Toronto, 850 (19.5%) to Ottawa, 320 (7.3%) to Vancouver/Victoria, and 208 (4.8%) to Montreal—Centre hospitalier de l'Université de Montréal. The Calgary site is new to GeoSentinel (as of 2012) and therefore did not contribute any cases during the study period. Major demographic variables for the cohort of 4365 travellers are summarized in Table 1. More than half (2418 [55.4%]) of the cohort was born in Canada (Table 1). Top countries of origin for individuals born outside of Canada were India (174 [4.0%]), China (91 [2.1%]), United States (75 [1.7%]), Philippines (75 [1.7%]), France (72 [1.6%]), Haiti (69 [1.6%]), United Kingdom (53 [1.2%]), Viet Nam (50 [1.1%]), and Somalia (47 [1.1%]).

**Table 1:** Demographic characteristics of 4365 returned travellers or new immigrants presenting to a CanTravNet site for care of a presumed travel-related illness, 2009–2011*

Characteristic	All travellers n = 4365	Purpose of travel †; no. (%) of travellers ‡					
		Tourism n = 2010	Immigration n = 876	Visit friends and relatives n = 500	Missionary, volunteer, researcher, aid n = 431	Business n = 359	Other§ n = 139
**Sex**							
Male	2026 (46.4)	836 (41.6)	492 (56.2)	243 (48.6)	159 (36.9)	220 (61.3)	54 (38.8)
Female	2337 (53.5)	1173 (58.4)	383 (43.7)	257 (51.4)	272 (63.1)	139 (38.7)	85 (61.2)
Unknown	2 (< 0.1)	1 (< 0.1)	1 (0.1)	0 (0)	0 (0)	0 (0)	0 (0)
**Age, yr, median (range)**	38 (0–95)	38 (0–89)	41 (1–95)	39 (0–88)	30 (2–85)	43 (11–83)	25 (13–74)
**Type of patient**							
Inpatient	224 (5.1)	60 (3.0)	64 (7.3)	53 (10.6)	17 (3.9)	19 (5.3)	10 (7.2)
Outpatient	4112 (94.2)	1950 (97.0)	810 (92.5)	447 (89.4)	414 (96.1)	339 (94.4)	129 (92.8)
Unknown	29 (0.7)	0 (0)	2 (0.2)	0 (0)	0 (0)	1 (0.3)	0 (0)
**Travel duration, d, median (range)**	20 (0–6632)	15 (0–3294)	NA	31 (0–2236)	37 (0–4104)	21 (0–1835)	62.5 (1–847)
**Pretravel medical encounter**							
Yes	1482 (34.0)	790 (39.3)	NA	104 (20.8)	304 (70.5)	180 (50.1)	89 (64.0)
No	1614 (37.0)	683 (34.0)	NA	289 (57.8)	41 (9.5)	97 (27.0)	19 (13.7)
Unknown	1269 (29.1)	537 (26.7)	NA	107 (21.4)	86 (20.0)	82 (22.8)	31 (22.3)
**Syndromic diagnoses**							
Gastrointestinal	1909 (43.7)	938 (46.7)	445 (50.8)	228 (45.6)	207 (48.0)	157 (43.7)	68 (48.9)
Dermatologic	642 (14.7)	488 (24.3)	52 (5.9)	52 (10.4)	50 (11.6)	49 (13.6)	17 (12.2)
Systemic febrile illness	470 (10.8)	198 (9.9)	50 (5.7)	112 (22.4)	62 (14.4)	67 (18.7)	20 (14.4)
Respiratory	235 (5.4)	92 (4.6)	82 (9.4)	24 (4.8)	23 (5.3)	26 (7.2)	10 (7.2)
**Geographic region of exposure**							
Sub-Saharan Africa	931 (21.3)	191 (9.5)	260 (29.7)	137 (27.4)	185 (42.9)	108 (30.1)	49 (35.3)
Caribbean	572 (13.1)	414 (20.6)	55 (6.3)	29 (5.8)	44 (10.2)	24 (6.7)	5 (3.6)
South Central Asia	516 (11.8)	174 (8.7)	125 (14.3)	129 (25.8)	34 (7.9)	30 (8.4)	24 (17.3)
Central America	484 (11.1)	372 (18.5)	12 (1.4)	25 (5.0)	45 (10.4)	18 (5.0)	12 (8.6)
South East Asia	389 (8.9)	167 (8.3)	143 (16.3)	33 (6.6)	12 (2.8)	29 (8.1)	5 (3.6)
South America	253 (5.8)	135 (6.7)	23 (2.6)	36 (7.2)	43 (10.0)	10 (2.8)	5 (3.6)
North East Asia	165 (3.8)	30 (1.5)	85 (9.7)	15 (3.0)	5 (1.2)	25 (7.0)	5 (3.6)
North America	140 (3.2)	94 (4.7)	6 (0.7)	5 (1.0)	2 (0.5)	16 (4.5)	6 (4.3)
Other||	349 (8.0)	132 (6.6)	115 (13.1)	51 (10.2)	14 (3.2)	25 (7.0)	11 (7.9)
Unknown	566 (13.0)	301 (15.0)	52 (5.9)	40 (8.0)	47 (10.9)	74 (20.6)	17 (12.2)
**Birth country**							
Canada	2418 (55.4)	1611 (80.1)	0 (0)	82 (16.4)	351 (81.4)	229 (63.8)	104 (74.8)
Outside Canada	1947 (44.6)	399 (19.9)	876 (100)	418 (83.6)¶	80 (18.6)	130 (36.2)	35 (25.2)
**Immigrant**							
Yes	1837 (42.1)	357 (17.8)	876 (100)	403 (80.6)	65 (15.1)	112 (31.2)	16 (11.5)
No or unknown	2528 (57.9)	1653 (82.2)	0 (0)	97 (19.4)	366 (84.9)	247 (68.8)	123 (88.5)
Delay to presentation, ** d, median (range)	30 (0–184)	28 (0–184)	78 (4–183)	35 (0–184)	28 (0–180)	30.5 (0–184)	29 (0–180)

* The cohort consisted of 3943 travellers with a definitive travel-related diagnosis, 363 with a non-travel-related diagnosis, and 59 with a diagnosis for which relation to travel could not be ascertained.

† Purpose of travel was available for 4315 of the 4365 members of the cohort.

‡ Except where indicated otherwise.

§ Includes students (n = 111), military personnel (n = 24), and medical tourists (n = 4); does not include those for whom reason for travel was unknown (n = 50).

|| Western Europe, Eastern Europe, the Middle East, North Africa, Australia/New Zealand, and Oceania.

¶ Among those born outside of Canada, people who travelled for the purpose of visiting friends and relatives were defined as immigrants who were ethnically and/or racially distinct from the majority population in their current country of residence and who returned to their homeland to visit friends and relatives. This group also included children of foreign-born parents (i.e., second-generation immigrants) who returned to their parents' homeland to visit friends and relatives.

** Interval between the end of travel and presentation at CanTravNet site.

**Figure 1:**
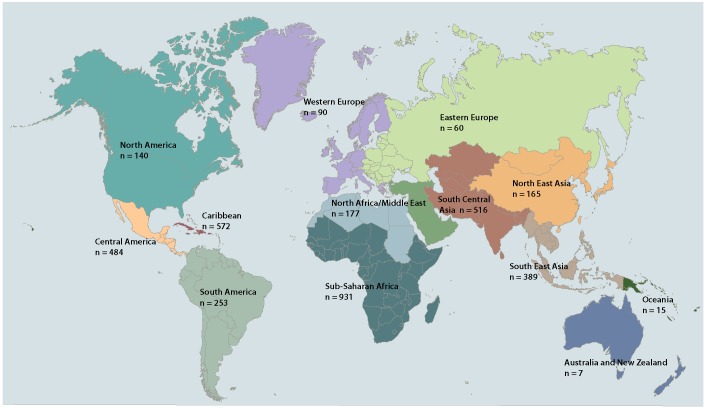
Regional exposure for the cohort of travellers. Base map adapted from vectorworldmap.com, version 2.2.

The non-immigrant travellers in the cohort (i.e., all members of the cohort except those who travelled for the purpose of immigration) for whom exposure country was known (n = 3 439) visited 154 different countries, most frequently India (287 [8.3%]), Mexico (231 [6.7%]), Cuba (153 [4.4%]), Dominican Republic (138 [4.0%]), Costa Rica (92 [2.7%]), the United States (73 [2.1%]), Ghana (72 [2.1%]), Thailand (71 [2.1%]), Peru (65 [1.9%]), and China (58 [1.7%]). The immigrant travellers in the cohort (i.e., those travelling for the purpose of i mmigration) (n = 8 76) e migrated f rom 1 13 d ifferent countries, the most frequent of which were China (66 [7.5%]), India (66 [7.5%]), Philippines (55 [6.3%]), Haiti (48 [5.5%]), Somalia (33 [3.8%]), Viet Nam (31 [3.5%]), Thailand (27 [3.1%]), Burundi (23 [2.6%]), Congo (23 [2.6%]), Cameroon (19 [2.2%]), and Myanmar (19 [2.2%]). Figure 1 depicts regional exposure for the cohort of travellers.

### Diagnoses

A total of 4774 travel-related diagnoses (4278 confirmed and 496 probable) were assigned to the 3943 ill returned travellers. Table 2 summarizes the top travel-related etiologic and syndromic diagnoses by travel reason. The most frequent travelrelated etiologic diagnoses for non-immigration travellers (n = 3115) were arthropod bite (127 [4.1%]), giardiasis (91 [2.9%]), malaria (77 [2.5%]), latent tuberculosis (73 [2.3%]), and strongyloidiasis (66 [2.1%]). Among those who travelled for immigration (n = 828), the most frequent travel-related etiologic diagnoses were latent tuberculosis (229 [27.7%]), chronic hepatitis B (n = 182 [22.0%]), active tuberculosis (97 [11.7%]), chronic hepatitis C (89 [10.7%]), and strongyloidiasis (41 [5.0%]). Common travel-related syndromic diagnoses in non-immigration travellers included chronic diarrhea (252 [8.1%]), acute diarrhea (243 [7.8%]), post-infectious irritable bowel syndrome (IBS) (232 [7.4%]), rash (125 [4.0%]), and skin and soft-tissue infections (110 [3.5%]). Cases of potentially serious imported infections, such as dengue fever (occurring in 61 ill returned travellers) and enteric fever due to *Salmonella enterica* serotype Typhi or Paratyphi (occurring in 36 ill returned travellers), were also common (Table 3).

**Table 2:** Top 10 syndromic and etiologic diagnoses by reason for travel among 3943 ill returned travellers with *definitive travel-related diagnoses*,* 2009–2011

Rank	Immigrants with travelrelated diagnosis n = 828	Travellers with travel-related diagnosis unrelated to immigration; no. (%) of travellers					
		All non-immigration travellers n = 3115	Tourism n = 1805	Visit friends and relatives n = 444	Missionary, volunteer, researcher, aid n = 416	Business n = 320	Other† n = 130
Total no. of travel-related diagnoses	1114	3660	2094	534	492	381	159
1	Latent TB 229 (27.7)	Chronic diarrhea 252 (8.1)	Acute diarrhea‡ 154 (8.5)	Malaria 34 (7.7) (*P. falciparum *23 [5.2])	Chronic diarrhea 47 (11.3)	Acute diarrhea‡ 27 (8.4)	PI-IBS 17 (13.1)
2	Chronic HBV 182 (22.0) (acute HBV 3 [0.4])	Acute diarrhea‡ 243 (7.8)	PI-IBS 151 (8.4)	Strongyloidiasis 25 (5.6)	Acute diarrhea‡ 37 (8.9)	Chronic diarrhea 27 (8.4)	Latent TB 10 (7.7)
3	Active TB 97 (11.7) (pulmonary TB 67 [8.1])	PI-IBS 232 (7.4)	Chronic diarrhea 148 (8.2)	Acute diarrhea‡ 18 (4.1)	PI-IBS 26 (6.3)	PI-IBS 25 (7.8)	Chronic diarrhea 10 (7.7)
4	Chronic HCV 89 (10.7)	Arthropod bite 127 (4.1)	Arthropod bite 102 (5.7)	Chronic diarrhea 17 (3.8)	Abdominal pain 20 (4.8)	Malaria 22 (6.9) (*P. falciparum *14 [4.4])	Viral syndrome no rash 7 (5.4)
5	Strongyloidiasis 41 (5.0)	Rash 125 (4.0)	Giardiasis 59 (3.3)	PI-IBS 13 (2.9)	Febrile illness < 3 wk duration 15 (3.6)	URTI 9 (2.8)	Skin and soft-tissue infection 6 (4.6)
6	Schistosomiasis 39 (4.7)	Skin and soft-tissue infection 110 (3.5)	Abdominal pain 56 (3.1)	Latent TB 13 (2.9)	Viral syndrome, no rash 13 (3.1)	Latent TB 8 (2.5)	Giardiasis 5 (3.8)
7	Filariasis 26 (3.1)	Giardiasis 91 (2.9)	Skin and soft-tissue infection 49 (2.7)	Abdominal pain 12 (2.7)	Giardiasis 13 (3.1)	Dengue fever 8 (2.5)	Acute diarrhea‡ 5 (3.8)
8	Hypertension 23 (2.8)	Malaria 77 (2.5) (*P. falciparum* 49 [1.6])	Viral syndrome, no rash 45 (2.5)	Viral syndrome, no rash 12 (2.7)	Dengue fever 10 (2.4)	Giardiasis 8 (2.5)	Active TB 4 (3.1) (pulmonary TB 3 [2.3])
9	Diabetes 15 (1.8)	Latent TB 73 (2.3)	Febrile illness < 3 wk duration 41 (2.3)	Chronic HBV 11 (2.5)	Latent TB 9 (2.2)	Strongyloidiasis 8 (2.5)	Dientamoebiasis 3 (2.3)
10	Anemia 14 (1.7)	Strongyloidiasis 66 (2.1)	Dientamoebiasis 35 (1.9)	Blastocystis 9 (2.0)	Strongyloidiasis 8 (1.9)	Febrile illness < 3 wk duration 8 (2.5)	Cutaneous leishmaniasis 3 (2.3)

HBV = hepatitis B virus, HCV = hepatitis C virus, P. falciparum = Plasmodium falciparum, PI-IBS = post-infectious irritable bowel syndrome, TB = tuberculosis, URTI = upper respiratory tract infection.

* For this table, only the 3943 people with a definitive diagnosis related to their travel (out of the 4365 returned travellers and new immigrants included in the study overall) were considered.

† Includes students (n = 103), military personnel (n = 24), and medical tourists (n = 3); does not include those for whom reason for travel was unknown.

‡ Includes acute bacterial, parasitic, and viral diarrhea, as well as acute diarrhea of unspecified etiology.

**Table 3:** Top diagnoses and source countries for specific etiologies within syndromic chief complaints among 3943 ill returned travellers with definitive travel-related diagnoses

Diagnosis	No. (%) of patients with chief complaint*	Total no. of patients in database with travelrelated diagnosis†	Top 3 source countries for diagnosis‡
**Chief complaint fever (n = 675)**			
Malaria	80 (11.9)	94	
*Plasmodium falciparum*	47 (7.0)	56	Ghana, Burkina Faso, Guinea (includes data for severe and cerebral malaria, as well as *P. falciparum* malaria)
Severe noncerebral	3 (0.4)	5	
Severe cerebral	3 (0.4)	3	
* Plasmodium vivax*	17 (2.5)	17	India, Honduras, Pakistan
* Plasmodium ovale*	5 (0.7)	5	Uganda, Malawi, Ghana (includes data for *P. malariae* malaria and unspecified malaria, as well as *P. ovale* malaria)
* Plasmodium malariae*	1 (0.1)	2	
Dengue fever	48 (7.1)	61	India, Indonesia, Nicaragua, Haiti
Active tuberculosis	47 (7.0)	123	India, China, Philippines
Pulmonary	34 (5.0)	82	
Extrapulmonary	13 (1.9)	41	
Enteric fever	28 (4.1)	33	India, Bolivia, Tanzania, Pakistan, Bangladesh
* Salmonella enterica* serotype Paratyphi	11 (1.6)	13	
Typhoid fever, unspecified	9 (1.3)	11	
* Salmonella enterica* serotype Typhi	8 (1.2)	9	
Upper respiratory tract infection	17 (2.5)	49	India, Mexico, Ghana
Pneumonia	16 (2.4)	23	Mexico, Canada, United States
Lobar	11 (1.6)	16	
Atypical	5 (0.7)	7	
Influenza-like illness	12 (1.8)	15	Tanzania, Panama, Brazil
Acute urinary tract infection	10 (1.5)	25	Mexico, India, Cameroon
Chikungunya fever	6 (0.9)	9	India, Indonesia, Malaysia
Brucellosis	6 (0.9)	7	India, Syria
Rickettsioses, spotted fever§	5 (0.7)	6	South Africa, Swaziland
**Chief complaint gastrointestinal (n = 1950)**			
Chronic diarrhea	254 (13.0)	254	Mexico, Cuba, India
Acute diarrhea||	235 (12.1)	241	India, Mexico, Cuba
Post-infectious irritable bowel syndrome	229 (11.7)	235	India, Mexico, Cuba, Dominican Republic
Giardiasis	84 (4.3)	96	India, Mexico, Costa Rica
Dientamoebiasis	59 (3.0)	62	Mexico, India, Thailand
Campylobacteriosis	22 (1.1)	24	Peru, India
Cryptosporidiosis, cyclosporiasis	16 (0.8)	17	Philippines, Mexico, India
Amoebiasis due to *Entamoeba histolytica*¶	11 (0.6)	12	India, Sri Lanka, Honduras
**Chief complaint dermatologic (n = 865)**			
Rash	128 (14.8)	138	Mexico, Cuba, Peru
Atopic dermatitis	19 (2.2)	21	
Contact dermatitis	21 (2.4)	21	
Drug reaction	4 (0.5)	8	
Photosensitivity reaction	11 (1.3)	12	
Unknown rash	51 (5.9)	54	
Urticarial	18 (2.1)	18	
Arthropod bite	123 (14.2)	128	United States, Cuba, Mexico
Insect**	99 (11.4)	104	
Tick or spider	24 (2.8)	24	
Skin and soft-tissue infection††	112 (12.9)	122	India, Cuba, Costa Rica
Cutaneous larva migrans	61 (7.1)	62	Jamaica, Mexico, Barbados
Animal bite‡‡	26 (3.0)	29	Thailand, India, Honduras
Cutaneous leishmaniasis	21 (2.4)	21	Syria, Libya, Costa Rica, Belize, Afghanistan
Marine envenomation	17 (2.0)	19	Cuba, United States, Mexico

* Percentages are calculated in relation to the category of chief complaint. An ill returned traveller could present with more than one chief complaint.

† Number of patients in the database who had the specific travel-related diagnosis, including those who did and those who did not have the corresponding chief complaint.

‡ Where 4 or 5 countries are listed, there was a 2-way or 3-way tie, respectively, for third place.

§ I ncludes infection with *Rickettsia africae, R. conorii*, and *R. rickettsii*.

|| Includes acute bacterial, parasitic, and viral diarrhea, as well as acute diarrhea of unspecified cause.

¶ Includes both intestinal and extraintestinal amoebiasis.

** Includes lice, fleas, true bugs, mosquitoes, flies, and midges.

†† Includes erysipelas, cellulitis, furunculosis, carbuncles, skin abscess, pyoderma, ecthyma, impetigo, and superficial fungal skin infections.

‡‡ Includes bites by cats, dogs, monkeys, and other animals.

Travel-related tuberculosis was a common diagnosis (n = 425), and the majority of cases (302 [71.1%]) were classified as latent, as indicated by a positive tuberculin skin test. The other types of tuberculosis documented among ill returned travellers were pulmonary (82 [19.3%]), extrapulmonary (31 [7.3%]), central nervous system or meningeal (7 [1.6%]), disseminated (2 [0.5%]), and multidrug resistant or extensively drug resistant (1 [0.2%]; acquired in Viet Nam).

A total of 348 cases of travel-related blood-borne and sexually transmitted infections were diagnosed in this group of ill returned travellers. This total included 200 cases of acute or chronic hepatitis B virus infection and 97 cases of chronic hepatitis C virus infection; 185 (92.5%) of the hepatitis B cases and 89 (91.8%) of the hepatitis C cases were diagnosed in ill returned travellers who had travelled for immigration (Table 2). There were 15 cases of HIV infection, of which 10 (67%) occurred in those travelling for the purpose of immigration.

Tuberculosis (both latent and active), hepatitis B virus infection, and hepatitis C virus infection were all diagnosed more frequently in those travelling for the purpose of immigration than in those travelling for other purposes (*p* < 0.001 for all comparisons). Individuals travelling for the purpose of visiting friends and relatives (n = 500 [11.6% of those with known reason for travel]) were over-represented among those diagnosed with malaria and enteric fever, compared with other illnesses (for malaria 34/94 [36.2%] v. 466/4221 [11.0%]; for enteric fever, 17/36 [47.2%] v. 483/4279 [11.3%]) (both *p* < 0.001). For cases of malaria, there was also overrepresentation (compared with other illnesses) from business travellers (22/94 [23.4%] v. 337/4221 [8.0%]) and males (62/94 [66.0%] v. 1964/4269 [46.0%]) (both *p* < 0.001). Of the 22 cases of malaria in business travellers, 15 (68%) had received a pretravel consultation, yet only 3 took appropriate malaria prophylaxis. Of 7 cases of malaria diagnosed in ill returned pediatric travellers, 5 (71%) were caused by *Plasmodium falciparum*, and all occurred in children travelling for the purpose of immigration (n = 5) or to visit friends and relatives (n = 2). Travellers with malaria were also more likely to require inpatient management of their illness (41/94 [43.6%]) than were those with other travel-related diagnoses (170/3849 [4.4%]).

The proportion of ill returned travellers who had been visiting friends and relatives and who required inpatient management of their travel-acquired illness was approximately double that of ill returned travellers who travelled for other reasons (*p* < 0.001) (Table 1).

People travelling to visit friends and relatives also had the lowest proportionate uptake of pretravel consultation among all ill returned non-immigrant travellers (*p* < 0.001) (Table 1). In addition, these individuals travelled for longer periods than those travelling for other reasons (31 v. 19 days; *p* < 0.001). And, as previously mentioned, although people travelling to visit friends and relatives constituted 11.6% of the cohort described here, they accounted for more than one-third of malaria cases and almost half of cases of enteric fever due to *S. enterica* serotype Typhi or Paratyphi.

The single case of measles in this study was imported by a person who travelled to India to visit friends and relatives. During our surveillance period, cases of highly communicable travel-acquired influenza (n = 18) and varicella (n = 1) were also reported, as was one case of Japanese encephalitis, which occurred in a tourist who went to Thailand.

Table 3 summarizes the top travel-related diagnoses and source countries among those ill returned travellers who presented for care complaining of fever, skin rash, or gastrointestinal symptoms. Table 4 summarizes the species of malaria, top countries of exposure, pretravel encounters, and prophylaxis status for all cases of malaria by purpose of travel.

**Table 4:** Cases of malaria among 3943 ill returned travellers with a travel-related diagnosis, by purpose of travel

Reason for travel	Total no. of cases	Type of malaria; no. of cases					Top 2 countries of exposure	Obtained pretravel advice	Received prophylaxis
		*P. falciparum*	Severe or cerebral malaria	*P. vivax*	*P. ovale*	*P. malariae* or unknown species			
All (n = 3943)	94	56	8	17	5	8	See Table 3	35	14
Tourism (n = 1805)	12	4	1	5	1	1	Honduras Uganda Burkina Faso*	5	4
Immigration (n = 828)	11	7	0	3	0	1	Liberia India	NA	1
Visit friends and relatives (n = 444)	34	23	2	6	2	1	India Cameroon	6	6
Missionary, volunteer, researcher, aid (n = 416)	10	7	1	0	1	1	Ghana Burkina Faso	7	1
Business (n = 320)	22	14	3	1	1	3	Ghana Guinea	15	3
Other † (n = 130)	5	1	1	2	0	1	India Burkina Faso Mali *	2	0

NA = not applicable, *P. = Plasmodium.*

* Two-way tie for second place.

† Includes students (n = 103), military personnel (n = 24) and medical tourists (n = 3).

Malaria and infection with blood-borne pathogens, such as hepatitis B virus and hepatitis C virus, were diagnosed more frequently in those who travelled to or emigrated from sub-Saharan Africa than in those who travelled to other regions (data not shown; *p* < 0.001 for all comparisons). Vaccine-preventable diseases, such as acute and chronic hepatitis B, hepatitis A, and Japanese encephalitis, were diagnosed more frequently in those who travelled to or immigrated from South East Asia and North East Asia (*p* < 0.001 for all comparisons). Dengue fever was diagnosed more frequently in those who travelled to the Caribbean or South East Asia (*p* = 0.003). Enteric fever was more likely to be acquired in South Central Asia than in other regions (*p* < 0.001). Only 9 cases of Chikungunya fever were diagnosed (Table 3).

## Interpretation

Analysis of surveillance data on ill returned travellers presenting to 1 of 5 CanTravNet sites between September 2009 and September 2011 revealed the spectrum of travel-acquired illness encountered at these sites. To date, this is the largest surveillance report on illness in Canadians travelling from abroad. Highlights of this analysis are presented in Box 1.

Box 1 **Highlights of returned Canadian travellers' surveillance data**

- The surveillance data reported here constitute a Canada-specific epidemiologic roadmap of diseases and syndromes acquired by a large group of international travellers and migrants.
- Fever in the returned traveller is a medical emergency because it may be due to a potentially serious and life-threatening infection such as malaria. Prompt medical attention is warranted.
- Those travelling to visit friends and relatives represent a specific group of at-risk travellers who are more frequently diagnosed with malaria and enteric fever due to *Salmonella enterica* serotype Typhi (also known as "typhoid fever") than with other illnesses.
- Highly feared travel-acquired illnesses such as Ebola hemorrhagic fever, Lassa fever, yellow fever, and meningococcal meningitis were not observed during the study period, but highly communicable, vaccine-preventable illnesses such as influenza, varicella, and measles were all imported during this period.
- Many travel-acquired infections are preventable by specific behaviours, vaccines, or chemoprophylactic medications, so interventions and policies that encourage uptake of pretravel advice should be promoted.
- Canadians who plan to travel internationally should seek a pretravel medical consultation at least 6 weeks before their trip, as recommended by the Public Health Agency of Canada.^13^

### Serious imported infections are common among ill returned Canadian travellers

Potentially life-threatening infections such as dengue, malaria, and enteric fever are commonly imported conditions with specific demographic and geographic preponderances. Chikungunya, which presents as a dengue-like illness, was rarely reported within this cohort, but it is an emerging arboviral infection of travellers to south Asia and the Indian Ocean islands,^14^ and has emerged more recently in the Caribbean. Ill returned travellers with malaria were proportionately more likely to require inpatient management than those with other diagnoses. Of the 94 cases of malaria in ill returned travellers, 60% were caused by *P. falciparum*, which can lead to severe and fatal disease. Moreover, 8.5% of travellers with malaria had complicated or cerebral malaria, which underscores the potential severity of this highly preventable illness among travellers, as outlined in the Canadian recommendations for the management of falciparum malaria in Canada.^15^ Of the 174 cases of imported severe or cerebral malaria reported in Canada by the Canadian Malaria Network between 2001 and 2012, 95% were due to *P. falciparum* (unpublished data [A.E.M.]). In addition, cases of severe or cerebral malaria have increased over time, from 11 in 2001 to 26 in 2012 (unpublished data [A.E.M.]). It should be noted that pretravel advice is not routinely covered by provincial health plans, nor are many travel medications such as malaria prophylaxis. Lack of coverage may be an important reason why certain groups of travellers have fewer pretravel encounters.

The relative risk of malaria among travellers is consistently highest in sub-Saharan Africa.^11^,^14^,^16^ We have confirmed that sub-Saharan Africa is the source region for most malaria among ill returned Canadian travellers, accounting for almost 77% of cases in this cohort. Of the 22 cases of malaria in business travellers, all but one were acquired in sub-Saharan Africa. In contrast, dengue was more often associated with travel to the Caribbean and South East Asia.^17^ Other potentially serious infections such as typhoid and paratyphoid fevers were more likely to be acquired in South Central Asia, a finding that supports previously published data.^10^,^18^,^19^

### Cosmopolitan and vaccine-preventable diseases were observed

Although serious and transmissible infections of particular public health importance (such as viral hemorrhagic fever due to Ebola or Lassa virus, anthrax, and meningococcal meningitis) were not observed in this cohort of ill returned travellers, other cosmopolitan and potentially vaccine-preventable infections were noted. Every year, sporadic cases of highly communicable yet vaccinepreventable infections such as measles, varicella, and influenza are imported by travellers. Since the mid-1990s, more than 80% of all cases of measles in the United States and Canada have been imported from measles-endemic countries or have been epidemiologically related to imported cases.^14^,^20^–^23^ A single case of measles imported to Quebec in 2011 led to a superspreading event with sustained transmission and 678 local cases, representing the largest measles epidemic in North America over the past decade.^23^ Thus, while emerging imported infections such as SARS (severe acute respiratory syndrome) and MERS (Middle Eastern respiratory syndrome) receive considerable public attention, other travel-acquired illnesses that are imported into North America also exact a heavy cumulative personal and public health toll.

### Travelling to visit friends and relatives is a risk factor for travel-acquired illness

Travelling for the purpose of visiting friends and relatives is a documented risk factor for the acquisition of travel-related illness, as people travelling for this reason tend to stay in local homes, travel for longer durations, and may fail to recognize the health risks inherent to travelling to their country of origin. 3,4,10,12,14,18 These travellers may also fail to recognize that immunity to diseases such as malaria wanes quickly after leaving an endemic area where repeated immune boosting may have occurred.^16^ It has previously been documented that 40% to more than 90% of imported cases of typhoid fever in North America occur in people who have travelled to visit friends and relatives.^19^,^24^ In the current study, double the proportion of people travelling to visit friends and relatives required inpatient management of their travel-acquired illness, and these people travelled for longer periods than ill returned people who travelled for other reasons, both of which highlight the unique nature of this particular type of traveller. In addition, this group had half the rate of pretravel encounters as other types of travellers. In 2010, people travelling to visit friends and relatives accounted for 17.4% of trips taken by Canadian travellers to overseas countries, with 1.83 million overnight visits; thus, the scale of travel to visit friends and relatives is substantial (*International travel survey: Canadian residents 2000–2010* [custom extract supplied by Statistics Canada]).

### Behaviour influences exposure risks

The behaviour of individual travellers dictates the potential for exposure to and acquisition of infectious diseases abroad.^25^–^29^ Travellers are much more likely to be exposed to blood and body fluids while travelling than when they are at home^29^ and are more likely to engage in risk-taking behaviours during which exposure to blood and body fluids may occur.^28^,^29^ The data reported here illuminate the frequency of blood-borne and sexually acquired illnesses in travellers, such as hepatitis B and HIV infection, which are chronic and expensive to manage. Also, given that some diseases such as hepatitis B virus infection have long incubation periods, clinicians need to be cognizant of such etiologies when symptoms arise even a long time after travel dates.

### Diarrheal illness is a common cause of morbidity among travellers

The most common and wellrepresented group of infectious illnesses among travellers are those borne by food and water.^30^ These illnesses predominate in the developing world for one main reason: inadequate sewage and sanitation systems. Use of nonpotable water for drinking or for washing fruits and vegetables, as well as contamination of hands with fecal matter, places travellers at risk for common illnesses such as bacterial gastroenteritis and parasitic causes of diarrhea and dysentery. A gastrointestinal chief complaint predominated in this cohort of travellers, with a full 49.5% presenting with gastrointestinal symptoms. Furthermore, chronic diarrhea, post-infectious IBS, acute unspecified diarrhea, acute bacterial diarrhea, and giardiasis were all among the top diagnoses reported. In particular, among tourists, missionaries, volunteers, researchers, aid workers, and other travellers, 4 to 6 of the top 10 diagnoses were related to gastrointestinal problems (Table 2).

Traveller's diarrhea, while usually self-limited, is known to trigger post-infectious IBS, a chronic and potentially debilitating condition, the median duration of which is approximately 2 to 3 years.^31^ The diagnosis of post-infectious IBS is one of exclusion, requiring elimination of other etiologies by stool microbiology and possibly more costly diagnostic interventions such as imaging and colonoscopy.^32^ Chronic diarrhea necessarily imposes a heavy burden on the Canadian health care system, given that approximately half of travellers to tropical and subtropical destinations experience infectious diarrhea while travelling,^33^–^35^ 10% of whom likely go on to experience post-infectious IBS.^36^

### Limitations

This analysis had several limitations. First, the population analyzed represents only those ill returned travellers who presented to a CanTravNet clinic; as such, our conclusions may not extend to all ill returned travellers. However, the top countries of exposure for non-immigrant travellers paralleled the top countries visited by travelling Canadians in general, with Mexico, Cuba, the Dominican Republic, and China included among the top 10 destinations for both this cohort and the general Canadian population (*International travel survey: Canadian residents 2000–2010* [custom extract supplied by Statistics Canada]). The top 3 source countries for new immigrants to Canada (the Philippines, China, and India)^37^ were also represented among the top 4 countries of emigration for immigrant travellers in this cohort. Second, travellers with mild or self-limited illnesses or illnesses with very short or very long incubation periods may have sought care in different settings. For example, our study did not capture illnesses for which care was sought during travel. Similarly, ill travellers returning from destinations perceived to be low risk may be underrepresented in the CanTravNet database. Third, data on inpatient versus outpatient management may be influenced by regional variations in management guidelines. Fourth, our data do not permit estimation of incidence rates or destination- specific numeric risks for particular diseases.^10^,^38^ Finally, intersite variation in screening protocols for new immigrants and refugees may have led to over- or under-contributions of particular diagnoses from individual sites. Nearly half of all cases (48%) were contributed by Montreal sites, which may have introduced bias because of interprovince variation in travel patterns.

### Conclusions

The impact and importance of travelacquired illness is considerable. At an individual level, it can lead to reduced work and school productivity, home convalescence, hospital admission, and potentially death, along with costs related to diagnostic tests and other medical visits that occur during the evaluation of such illnesses. At a population level, the potential for travellers to import public health threats, such as measles, sexually transmitted infections, and blood-borne and vector-borne diseases, is substantial. Accurate knowledge of the health problems faced by international travellers in different geographic destinations provides a robust evidence base for physicians to deliver effective preventive advice, immunizations, and prophylactic medications to travellers. This profile further informs post-travel diagnosis and therapy, as well as prioritization of pretravel intervention strategies for the most significant illnesses. The evidence presented here contributes to program-level decisions of the Public Health Agency of Canada related to provision of travel health information and prevention and control of infectious diseases. Specifically, it can inform decisions about allocation of resources to address certain at-risk populations such as those travelling to visit friends and relatives and travellers to specific destinations. It also contributes to the Agency's situational awareness and response to emerging and ongoing outbreaks and enhances the recommendation statements of the Agency's expert advisory committee, CATMAT. In this way, the Canada-specific surveillance data reported here should inform public health policy and strategic initiatives related to defining, monitoring, and preventing travelacquired illness in Canadians.
